# Pericytes are protective in experimental pneumococcal meningitis through regulating leukocyte infiltration and blood–brain barrier function

**DOI:** 10.1186/s12974-023-02938-z

**Published:** 2023-11-17

**Authors:** Nina C. Teske, Susanne Dyckhoff-Shen, Paul Beckenbauer, Jan Philipp Bewersdorf, Joo-Yeon Engelen-Lee, Sven Hammerschmidt, Roland E. Kälin, Hans-Walter Pfister, Matthijs C. Brouwer, Matthias Klein, Rainer Glass, Diederik van de Beek, Uwe Koedel

**Affiliations:** 1 Department of Neurology, LMU University Hospital, LMU Munich, Munich, Germany; 2 ESCMID Study Group for Infections of the Brain, Basel, Switzerland; 3 Department of Neurology, Amsterdam UMC, University of Amsterdam, Amsterdam Neuroscience, Amsterdam, The Netherlands; 4 Department of Neurosurgery, LMU University Hospital, LMU Munich, Munich, Germany; 5 Department of Medicine, Memorial Sloan Kettering Cancer Center, New York, NY USA; 6 Department Genetics of Microorganisms, Interfaculty Institute of Genetics and Functional Genomics, University of Greifswald, Greifswald, Germany; 7 Neurosurgical Research, Department of Neurosurgery, LMU University Hospital, LMU Munich, Munich, Germany; 8 Walter Brendel Center of Experimental Medicine, Faculty of Medicine, LMU Munich, Munich, Germany

**Keywords:** Pericytes, Pneumococcal meningitis, Blood–brain barrier, *Streptococcus pneumoniae*

## Abstract

**Background:**

Brain pericytes participate in the regulation of cerebral blood flow and the maintenance of blood–brain barrier integrity. Because of their perivascular localization, their receptor repertoire, and their potential ability to respond to inflammatory and infectious stimuli by producing various cytokines and chemokines, these cells are also thought to play an active role in the immune response to brain infections. This assumption is mainly supported by in vitro studies, investigations in in vivo disease models are largely missing. Here, we analysed the role of brain pericytes in pneumococcal meningitis, in vitro and in vivo in two animal models of pneumococcal meningitis.

**Methods:**

Primary murine and human pericytes were stimulated with increasing concentrations of different serotypes of *Streptococcus pneumoniae* in the presence or absence of Toll-like receptor inhibitors and their cell viability and cytokine production were monitored. To gain insight into the role of pericytes in brain infection in vivo, we performed studies in a zebrafish embryo model of pneumococcal meningitis in which pericytes were pharmacologically depleted. Furthermore, we analyzed the impact of genetically induced pericyte ablation on disease progression, intracranial complications, and brain inflammation in an adult mouse model of this disease.

**Results:**

Both murine and human pericytes reacted to pneumococcal exposure with the release of selected cytokines. This cytokine release is pneumolysin-dependent, TLR-dependent in murine (but not human) pericytes and can be significantly increased by macrophage-derived IL-1b. Pharmacological depletion of pericytes in zebrafish embryos resulted in increased cerebral edema and mortality due to pneumococcal meningitis. Correspondingly, in an adult mouse meningitis model, a more pronounced blood–brain barrier disruption and leukocyte infiltration, resulting in an unfavorable disease course, was observed following genetic pericyte ablation. The degree of leukocyte infiltration positively correlated with an upregulation of chemokine expression in the brains of pericyte-depleted mice.

**Conclusions:**

Our findings show that pericytes play a protective role in pneumococcal meningitis by impeding leukocyte migration and preventing blood–brain barrier breaching. Thus, preserving the integrity of the pericyte population has the potential as a new therapeutic strategy in pneumococcal meningitis.

**Supplementary Information:**

The online version contains supplementary material available at 10.1186/s12974-023-02938-z.

## Introduction

Pneumocoocal meningitis (PM) is a severe infection affecting the protective lining of the brain and spinal cord caused by *Streptococcus pneumoniae*. Despite major advances in therapy over the past decades, the disease remains a life-threatening condition, with case fatality rates ranging from 10% to 30% [1, 2]. Furthermore, over 50% of survivors of PM suffer from persistent neurologic deficits, including cognitive impairment [1, 2]. The inflammatory response triggered by pneumococcal meningeal infection plays a crucial role in the development of brain damage and unfavorable clinical outcomes [1, 3]. There are still substantial gaps in our knowledge about how this inflammatory response is initiated and controlled [1, 2, 4].

Macrophages, the predominant immune cells in the meninges, are believed to orchestrate the meningeal defense against the invading pneumococci through releasing chemokines that recruit neutrophils and monocytes from the periphery [4]. These recruited cells contribute to the clearance of bacteria [4]. While this concept has been accepted widely [1, 4], there is still uncertainty regarding its definitive proof. While a recent study observed a marked reduction of recruited immune cells in the meninges as a result of clodronate-induced depletion of resident macrophages [5], two earlier studies found no or only mild effects of this depletion approach on the amount of inflammation [6, 7]. Our own experimental findings corroborated the latter studies [8], indicating that macrophage depletion alone is insufficient to completely suppress the immune reaction despite its high effectiveness. This suggests the involvement of other local cell types, in addition to macrophages, in mounting the host defense against bacterial invasion.

Pericytes emerged as potential candidates to be involved in the process of central nervous system (CNS) infection. First, their perivascular localization makes them ideally situated to act as immune gatekeepers [9–13]. Second, possess pattern recognition receptors (PRRs), including Toll-like receptors (TLRs) and non-TLR–PRR, enabling them to sense diverse bacterial pathogen-associated molecular patterns (PAMPs) [14]. Third, pericytes can respond to exposure to PAMPs or inflammatory mediators by releasing selected sets of cytokines and chemokines, such as interleukin (IL)-6, the C–C motif chemokine ligand 2 (CCL2), and the C–X–C motif chemokine ligand 1 (CXCL1) that may help to attract circulating leukocytes to the site of infection/inflammation [15–17]. Fourth, pericytes may aid leukocyte migration by expressing adhesion molecules, such as intercellular adhesion molecule-1 (ICAM-1) as well as matrix metalloproteinases [18]. Fifth, pericytes can internalize a range of matter entering a breached blood–brain barrier (BBB) through receptor-mediated endocytosis or nonspecific pinocytosis, and possess numerous lysosomal granules suggesting that they are structurally equipped to degrade internalized material [19]. Furthermore, pericytes play an important role in the integrity of the BBB [20–22], which is essential for preventing the entry of harmful substances, including pathogens, into the CNS. So far, little is known about the role of pericytes in bacterial CNS infectious diseases.

In in vitro BBB models, pericyte dysfunction and damage, leading to barrier disruption, have been described after challenge with the meningeal pathogens *Escherichia coli* and *Haemophilus influenzae* [23, 24]. When challenged with *S. pneumoniae*, pericytes enhance neutrophil transmigration across an in vitro endothelial barrier, although the barrier’s permeability remains unchanged [25]. The upregulation of neutrophil chemokines by the pericytes seems to be driven by paracrine signalling from neighbouring macrophages rather than by direct bacterial influence [25]. However, it remains uncertain whether these findings accurately represent the events occurring during bacterial CNS infections in vivo, as the role of pericytes seems to be highly context-dependent and organ-specific [11].

In the current study, we analysed the role of brain pericytes in PM, in vitro and in vivo in two animal models of PM.

## Materials and methods

### Bacterial culture conditions

*S. pneumoniae* wild-type (wt) strains 6B, 7F, and D39 as well as its isogenic pneumolysin (PLY)-deficient mutant D39Δ*ply* were cultured on Columbia blood agar plates (Oxoid, 5% sheep blood), resuspended and grown in Todd–Hewitt broth supplemented with 0.5% (w/v) yeast extract (THY; Carl Roth, X936.1) until midlogarithmic phase, and stored at − 80 °C in THY supplemented with 10% glycerol. Bacterial concentrations were quantified by measuring optical density (NanoPhotometer^®^ P-Class, Implen, Germany) and by counting colony forming units (CFU). For infection experiments, frozen vials of D39 were thawed and diluted with phosphate buffered saline (PBS, #D8537, Sigma-Aldrich, Germany) to a concentration of 10^7^ cfu/ml. For in vitro experiments, *S. pneumoniae* strains were thawed and diluted with a 1:10 v/v solution of PBS and penicillin/streptomycin (P/S; ThermoFisher, Germany, # 10378016) to a stock concentration to 2 × 10^8^ cfu/ml.

### Primary murine and human brain pericytes

Murine brain pericytes were isolated and cultured from C57BL6/n brains as described previously [26]. Human brain pericytes were obtained from ScienceCell Research Laboratories and cultured according to the manufacturer’s instructions (#1200; www.sciencellonline.com/human-brain-vascular-pericytes). To check the purity of our murine cultures, we performed RT-PCR analyses (see description below) using PrimePCR™ SYBR^®^ Green assays for known pericyte markers (PDGFRβ, CD13, Vcan, Acta2) and macrophage markers (Itgam, Cx3Cr1, Mrc1, Cd36). In these analyses, we consistently found expression of pericyte markers but not of macrophage markers. Furthermore, we analyzed the expression of macrophage markers CD11B, Cx3Cr1, Mrc1, CD36, CD163, and TREM2 in human pericytes compared to their PDGFR expression, both before and after stimulation with conditioned medium, using RT-PCR.

### Cell-culture experiments

Murine and human primary brain pericytes were challenged with different pneumococcal serotypes (multiplicity of infection [MOI] = 2.5, 10, 40, 160), namely, the serotype 6B, 7F, 2, and a pneumolysin (PLY)-deficient isogenic serotype 2 mutant; the serotype selection was based on known differences in their pathogenic potency [27–29]. All serotypes were lysed with P/S 1 h before their use. This eliminated possible effects of serotype differences in growth rate and ensured PAMP release. All experiments were carried out in the respective culture media containing 1% heat-inactivated, fetal bovine serum (ThermoFisher, # 10082147) and 1% P/S. To define the role of TLR signalling in pericyte and *S. pneumoniae* interaction, mouse and human pericytes were treated with a neutralizing antibody directed against TLR2 (T2.5; 25 µg/ml, #HM1054, Hycult Biotech, Uden, the Netherlands), the well-known endosomal TLR inhibitor chloroquine (CQ; 20 µg/ml, #C6628, Sigma-Aldrich, St. Louis, Missouri, USA), the TLR4 inhibitor TAK-242 (5 µM, #TAK-242, Sigma-Aldrich, St. Louis, Missouri, USA), and combinations of these drugs as well as the nuclear factor 'kappa-light-chain-enhancer' of activated B-cell (NF-κB) inhibitor parthenolide (5 µM, #70080, Cayman Chemical, Ann Arbor, Michigan, USA) prior to pneumococcal exposure (serotype 2, MOI = 40). To determine the influence of paracrine factors from macrophages on pericyte behavior, human brain pericytes were either exposed to conditioned media (at a 1.10 dilution with normal cell-culture medium) obtained from Tohoku Hospital Pediatrics-1 (THP-1) macrophages differentiated from wild-type (Wt), TLR2-deficient, Apoptosis-associated speck-like protein containing a caspase recruitment domain (ASC)-deficient, or NOD-, LRR- and pyrin domain-containing protein 3 (Nlrp3)-deficient THP-1 monocytic cells (all from Invivogen) with phorbol 12-myristate 13-acetate (PMA) or to conditioned media from differentiated Wt THP-1 macrophages in the absence or presence of inhibitors of the IL-1 signaling pathway (namely, the caspase-1 inhibitor VX-765, 100 µM, and the Nlrp3 inhibitor MCC950, 10 µM, both from Selleckchem, S2228 and S8930). At 6 h after exposure, cell-culture supernatants were sampled for cytokine analysis using proteome profiler assays (mouse proteome profiler array #ARY006 from R&D Systems and human C3 array from Raybiotech, #AAH-CYT-3) and selected cytokine ELISAs (DuoSet™ ELISA Kits from R&D Systems, USA, DY206 and DY406) as well as for cell viability determinations (LDH Cytotoxicity Assay Kit from Biovision, #K311-400).

Proteome profiler assay membranes were visualized using enhanced chemiluminesence (Femtomax-110; Rockland, USA) with an UVP Chemidoc-IT Imaging system (UVP, UK). The images were analyzed using ImageJ software (NIH, USA). To standardize the results, the optical densities (ODs) of each spot were expressed as a percentage of the average optical densities of the positive controls on each membrane. For the sake of specificity, the test's sensitivity was limited by disregarding relative expression levels below 5% of the positive controls. Proteins showing a more than twofold difference in expression levels between experimental groups were considered to be differentially expressed. All cell-culture experiments were carried out (at least) in duplicates at least for three times.

### Zebrafish model of pneumococcal meningitis

We used our established zebrafish model of PM [30, 31]. Wild-type and *TgBAC(pdgfrb:EGFP/Tg(fli1a:Myr-mCherry)* zebrafish adults (eggs kindly provided by Naoki Mochizuki and Shigetomo Fukuhara, Osaka, Japan) were kept at 26 °C in aerated 5 L tanks with a 14/10 h light/dark cycle as previously described [30–32]. Zebrafish embryos were collected within the first hour postfertilization (hpf) and then kept at 28 °C in E3 medium (5.0 mM NaCl, 0.17 mM KCL, 0.33 mM CaCl·2H_2_O, 0.33 mM MgCl_2_·7H_2_O). Twenty-four hours later embryos were mechanically dechorionated. Prior to injection, 2 day postfertilization (dpf) embryos were anaesthetized in 0.02% *(w/v)* buffered 3-aminobenzoic acid methyl ester (pH 7.0) (Tricaine; Sigma-Aldrich, #A5040, Sigma-Aldrich, St. Louis, Missouri) and then individually infected by microinjection with 1 nl of *S. pneumoniae* (serotype 2 D39 strain, 1900 and 2200 cfu diluted in 0.25% sterile phenol red solution (#P0290, Sigma-Aldrich, St. Louis, Missouri) in wt and *TgBAC(pdgfrb:EGFP/Tg(fli1a:Myr-mCherry)* embryos, respectively) in the hindbrain ventricle as described elsewhere [33]. Controls received 1 nl of 0.25% sterile phenol red solution. All experimental procedures comply with European animal welfare regulations.

### Survival experiments in infected zebrafish embryos

After hindbrain injection, wt and *TgBAC(pdgfrb:EGFP/Tg(fli1a:Myr-mCherry)* (tg) zebrafish embryos were kept in 6-well plates at 28 °C with 20 embryos per group in each well. The mortality rate was determined by monitoring live and dead embryos (death was defined as that do not respond to tail touches nor have a beating heart) at fixed timepoints (24, 48, 72 h post-injection; hpi). To assess the effect of pericyte depletion on survival, infected zebrafish embryos were treated orally with the PDGFRβ inhibitor AG1296 (Abcam; 20 µM, #ab141170, Abcam, Cambridge, Great Britain) in 1% Dimethylsulfoxid (DMSO) or 1% DMSO alone (vehicle control, 20 µM, #8418, Sigma-Aldrich, St. Louis, Missouri, USA) by adding the compounds to E3 medium for the whole infection course with refreshment of the E3 medium and compound every 24 h [34]. All experiments were performed in triplicate with 20 embryos per group.

### Histopathological analysis of zebrafish embryos

For histopathological analysis, wt zebrafish embryos were anaesthetized with tricaine (#A5040, Sigma-Aldrich, St. Louis, Missouri), fixated in 4% paraformaldehyde (Sigma-Aldrich, St. Louis, Missouri, USA) in phosphate buffered saline (PBS, DPBS, #0303, ScienCell, San Diego, Kalifornien, USA), embedded in paraffin and sectioned sagittally in 4 µm thickness. The section were mounted on glass slides and stained with haematoxylin and eosin. The stained slides were scanned with a Menari D-SIGHT *fluo* scanner (Florence, Italy) at × 100 magnification with oil immersion for histopathological evaluation. For the histopathological analysis at least five fish per group were evaluated.

### Fluorescence imaging of zebrafish embryos

Tg zebrafish embryos treated with 1-phenyl-2-thiourea (200 µM, #103-85-5, Sigma-Aldrich, St. Louis, Missouri, USA) between 8 and 48 hpi to prevent pigmentation were anaesthetized with tricaine 48 h after infection and fixated for 2 h in 4% paraformaldehyde in PBS. Subsequently, the embryos were washed with PBS and stored in a fridge for a maximum of 1 day. For optimal imaging, embryos were embedded in 1.5% low-melting-point agarose dissolved in PBS in an open uncoated 8-well microscopy µ-Slide (http://ibidi.com). Confocal images were generated with a Leica TCS SP8 Confocal Microscope. Leica Application Suite X and ImageJ software were used to process the confocal images, specifically for brightness/contrast enhancements as well as for creating merged images. For the fluorescence imaging at least five fish per group were evaluated.

### Mouse model of pneumococcal meningitis

We used a well-characterized mouse model of PM [35, 36]. Briefly, mice were weighed, their body temperature was measured using a rectal temperature probe, and their motor activity was assessed using the open field test (OFT). Subsequently, a physical examination was conducted (including clinical scoring). Clinical scoring comprised a beam walk test, a postural reflex test, presence/absence of pilo-erection, reduced vigilance, and/or seizures. The maximum clinical score was 13 indicating moribund mice that had to be euthanized due to ethical considerations, whereas a score of 0 defined uninfected healthy mice. Following this, the mice were subcutaneously treated with buprenorphine (WDT, #02540:Germany) for analgesia; buprenorphine was reapplied every 8 h to maintain its analgesic effect. One hour later, bacterial meningitis was induced through a transcutaneous puncture of the cisterna magna and the injection of 15 µl of 10^7^ colony forming units (cfu) per ml *S. pneumoniae* type 2 (D39 strain, dissolved in PBS) using an Omnican^®^ 100 insulin syringe under a short-term anesthesia with isoflurane. The mice were returned to their cages and reevaluated clinically upon awakening. Clinical score values were determined at the timepoints of 18 h (time of ceftriaxone administration), 24 h, and 42 h post-infection. At the end of the experiment, mice were anaesthetized with ketamine/xylazine. After local anesthesia with lidocaine, a skin incision was made to expose the skull cap. Thereafter, a catheter was inserted into the cisterna magna through a hole drilled at the caudal end of the occipital bone to collect cerebrospinal fluid (CSF) samples for white blood cell (WBC) counts. Blood samples were withdrawn to determine bacterial titers. Subsequently, animals were perfused transcardially with ice-cold heparinized PBS. The brain was removed, the cerebellum dissected and homogenized in 1 ml sterile PBS for determination of bacterial titers. The remaining brain tissue was frozen immediately in tissue freezing medium (Leica Biosystems) for cryosectioning and subsequent histopathological and molecular biological analyses.

### Genetic cell lineage ablation model

To generate a pericyte ablation model, we crossed the two mouse strains PDGFRB::creER2 (expressing tamoxifen-inducible Cre recombinase Cre^ERT2^ under the PDGFRB-promotor; JAX stock # 030201) [37] and R26-iDTA (expressing an attenuated form of the diphtheria toxin‐A chain after cre‐induced recombination in the Rosa26 locus; (JAX stock # 010527) [38] to generate PDGFRB::creER2-iDTA mice. As described previously [39], cre-mediated recombination, leading to DTA-mediated ablation of PDGFRβ-expressing cells, was induced by intraperitoneal injection of 75 mg/kg body weight tamoxifen (TAM; Sigma, T5648; dissolved in corn oil) every 24 h during 3 consecutive days starting 4 days before meningitis induction. PDGFRB::creER2-iDTA treated with corn oil as well as TAM- or corn oil (CO)-treated C57BL/6 mice were applied as control groups.

### Experimental groups in the mouse model

For the investigations, a total of 61 mice of both sexes (aged 12–20 weeks, weighing 20–30 g) were used. Mice were distributed among the experimental groups (in an age- and sex-matched manner) as follows: group I) C57BL/6 wt mice injected i.c. with PBS (controls; *n* = 4); groups II and III) C57BL/6 wt mice pre-treated with CO or TAM and injected i.c. with *S. pneumoniae* (*n* = 11, each group); groups IV and V) PDGFRB::creER2-iDTA mice pre-treated with CO or TAM and injected i.c. with *S. pneumoniae* (*n* = 11 and *n* = 12, respectively). For additional examinations of BBB permeability using the Evans-Blue method, another 12 mice were used, with four animals in each groups which were the following: uninfected, CO-pretreated, infected, CO-pre-treated PDGFRB::creER2-iDTA mice, and infected, TAM-pretreated PDGFRB::creER2-iDTA mice (groups VI–VIII).

### Determination of bacterial titers in blood and brain

Blood samples and cerebellar homogenates were diluted serially in sterile saline, plated on blood agar plates, cultured for 24 h at 37 °C with 5% CO_2_, and colonies thereon were counted out.

### Analysis of cerebral bleeding

Frozen brains were cut into 10 µm thick coronal sections on a cryostat. Beginning from the anterior parts of the lateral ventricles, ten serial sections were photographed with a digital camera in 0.3 mm intervals throughout the ventricle system. Haemorrhagic spots were counted and total bleeding area on each slice was determined (ImageJ, NIH).

### Immunohistochemical analysis of murine brains

Ten-µm-thick coronal brain sections containing the lateral ventricles and hippocampal tissue were either stained with a rat anti-mouse monoclonal antibodies directed against mouse PDGFRβ (AF1042, R&D Systems), CD13 (MCA2183GA, Bio-Rad) or the fibroblast marker ER-TR7 (sc-73355, Santa Cruz). After quenching endogenous peroxidase activity with 0.3% methanolic hydrogen peroxide and blockage of non-specific binding by 10% normal rabbit serum, brain sections were incubated with the PDGFRβ antibody, anti-CD13 antibody or the anti-TR7 antibody overnight at 4 °C, namely, in the following dilutions: 1:80, 1:100 and 1:80, respectively. Labelled cells were visualized using biotinylated rabbit anti-rat IgG at a 1:200 dilution, followed by horseradish peroxidase-conjugated streptavidin and then 3,3-diaminobenzidine as a chromogen (all from Vector Laboratories, Burlingame, USA). After counterstaining with Mayer’s hematoxylin solution, tissue sections were examined using an Olympus BX51 microscope and images captured with a cooled Moticam 5000 video camera (Moticam, Germany) connected to a PC.

### mRNA expression analyses of murine brains and cultured pericytes

Total RNA from murine brain sections as well as cultured pericytes was extracted using Aurum Total RNA Mini Kit (Bio-Rad, 7326820) according to manufacturer’s instructions. Reverse transcription into cDNA was achieved using the iScript cDNA Synthesis Kit (Bio-Rad, 1708890) according to manufacturer’s instructions. Quantitative PCR (qPCR) was performed on a qTower3 Real-Time PCR Detection System (Analytikjena) using the SsoAdvanced Universal Probes Supermix (Bio-Rad, 1725280) and PrimePCR™ SYBR^®^ Green assays () and a custom-made PrimePCR™ SYBR^®^ Green array containing 25 inflammatory cytokines and receptors, and 2 different housekeeping genes detailed on Additional file 6**:** table. GAPDH was used as reference gene. The custom-made array was performed in duplicate with pooled cDNA from randomly selected 4 mice per group.

### Evaluation of BBB permeability by an Evans blue assay

One hundred µl of a 2% solution of Evans Blue (EB; Sigma Aldrich, E2129) in PBS was injected intraperitoneally and allowed to circulate for 2 h. After anesthesia, blood samples were collected by cardiac puncture in a heparinized syringe and then centrifuged. Thereafter, mice were transcardially perfused with 20 ml of ice-cold heparinized PBS. The brains were removed, homogenized in 1 ml of PBS, and centrifuged. The respective supernatants were collected and diluted serially in PBS. To each sample an equal amount of 50% trichloroacetic acid (TCA) was added, incubated over night at 4 °C and then centrifuged. EB levels were measured by spectrophotometer at 610 nm and quantified according to a standard curve [40].

### Statistical analysis

Prior to project implementation, a sample size planning was carried out under the supervision of a statistician, and the test methods to be used for statistical analysis were established. Statistics and graphs were generated with GraphPad Prism software. The principal statistical test was an ANOVA with Tukey’s multiple comparisons test or a log-rank test (Mantel) for survival. Differences were considered significant at *P* values < 0.05. Data are given as mean ± standard deviation.

### Study approval

This study was carried out in accordance with the recommendations in the Guide for the Care and Use of Laboratory Animals (National Research Council, USA) and with the German Animal Protection Act. The study protocol was approved by the Committee on the Ethics of Animal Experiments of the Government of Upper Bavaria (Permit numbers 55.2-1-54-2532.Vet_02-18-169).

### Data availability

Data available on request from the authors.

## Results

### Human and murine brain pericytes release selected cytokines upon *S. pneumoniae* challenge

**Fig. 1:**
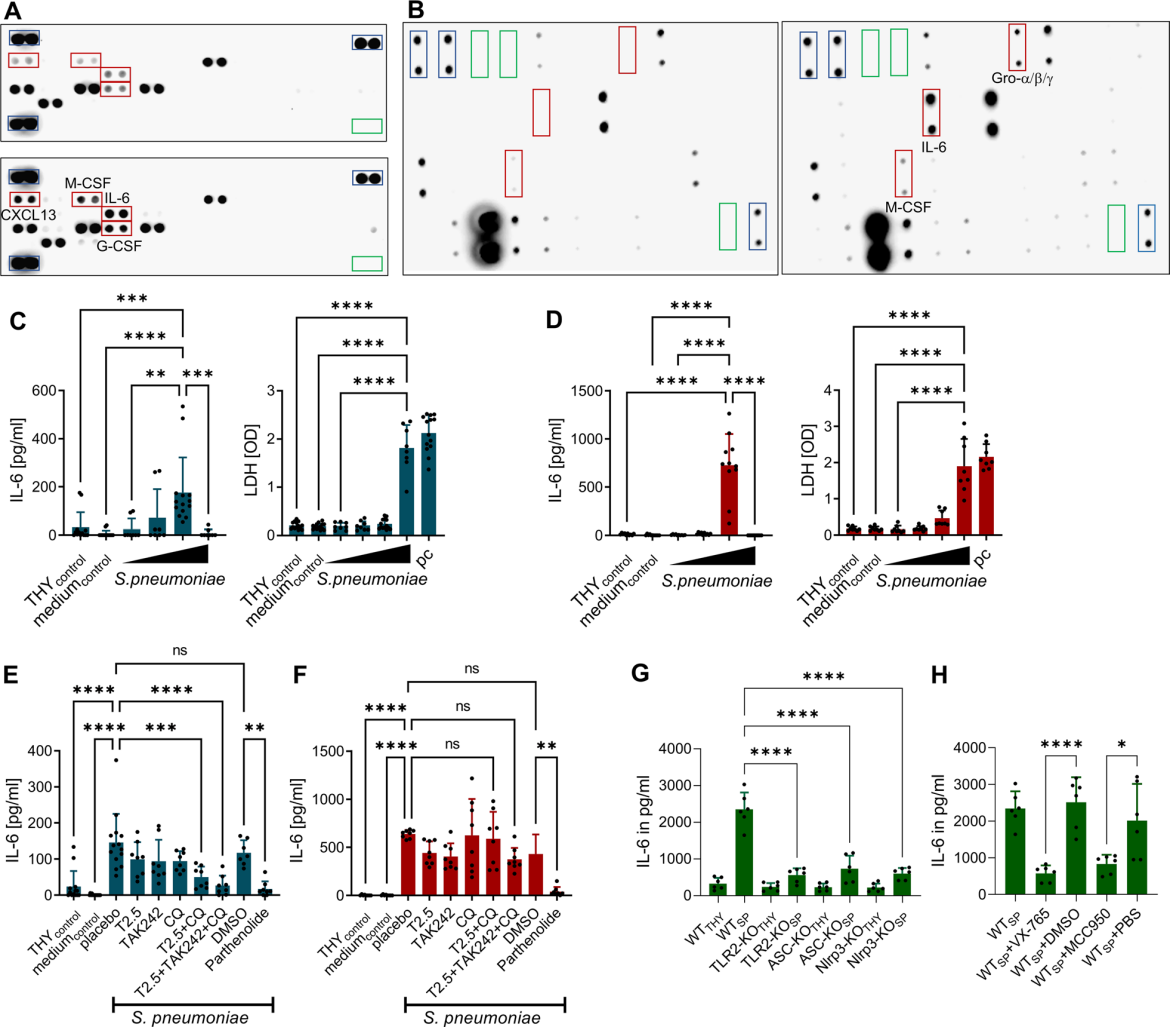
Human and murine brain pericytes release selected cytokines upon *S. pneumoniae* challenge. Protein array analyses of cell-culture supernatants obtained from murine (**A**) and human (**B**) primary brain pericytes stimulated with *S. pneumoniae* (MOI = 40) or its vehicle for 6 h. The differentially expressed proteins are outlined with red rectangles, the positive controls with blue rectangles and the negative controls with green rectangles. IL-6 concentrations (determined by ELSA) in cell-culture supernatants of murine (blue bars; **C**) and human (red bars; **D**) primary brain pericytes 6 h after exposure to increasing concentrations of antibiotic-lysed serotype 2 *S. pneumoniae* (MOI = 2.5, 10, 40, 160). THY (= Todd–Hewitt broth supplemented with 0.2% yeast extract, used for culturing *S. pneumoniae*) and cell-culture medium served as negative controls. Effect of various anti-TLR antagonists (T2.5 = neutralizing antir-TLR2 antibody; TAK242 = a TLR4 antagonist; CQ = chloroquine = an endosomal TLR antagonist) and the NF-κB inhibitor parthenolide on *S. pneumoniae* (MOI = 40)-induced IL-6 release from murine (**E**) and human (**F**) primary brain pericytes. Response of human brain pericytes to conditioned media (green bars) from wild-type (WT), TLR2-deficient, ASC-deficient, and Nlrp3-deficient THP-1 cells stimulated with either THY or *S. pneumoniae* (MOI = 80; **G**). Response of human brain pericytes to conditioned media from *S. pneumoniae*-stimulated WT THP-1 treated either with the caspase-1 inhibitor VX-765, the Nlrp3 inhibitor MCC950, or its vehicles (DMSO or PBS, **H**). Data are given as individual values as well as means ± SD. *P < 0.05, **P < 0.01, ***P < 0.001, ****P < 0.0001, using ANOVA with Tukey’s multiple comparisons test

We conducted a protein array analysis to investigate the response of cultured primary brain pericytes from mice and humans to *S. pneumoniae* exposure. In murine brain pericytes, we observed a more than twofold increase of 4 out of 40 cytokines, namely, CXCL13 (optical densities in % of positive controls: 14 vs 42 in control vs SP-stimulated cells), M-CSF (9 vs 33), G-CSF (19 vs 57), and IL-6 (20 vs 43) (Fig. 1A). Human pericytes stimulated by *S. pneumoniae* released three cytokines, namely, Gro-α/β/γ, M-CSF, and IL-6 (optical densities in % of positive controls in control vs SP-stimulated cells: 5 vs 25, not detectable vs 62, not detectable vs 169, respectively); no increase in G-CSF release was observed (Fig. 1B). The increase in IL-6 protein levels occurred in both species, leading us to use IL-6 as a surrogate marker in further analysis. We found that the release of IL-6 in both human and murine cells was independent of the pneumococcal serotype (albeit with differences in the dependence to concentrations; Additional file 1: Figure S1), but possibly dependent on the presence of the toxin pneumolysin (Additional file 1: Figure S1). In addition, treatment with the NF-κB inhibitor parthenolide prevented *S. pneumoniae*-induced IL-6 production from brain pericytes in both species. However, only murine brain pericytes exhibited recognition of *S. pneumoniae* through TLR-2 and endosomal TLRs (Fig. 1E, F). Furthermore, when exposed to high pneumococcal concentrations, both human and murine brain pericytes released significant amounts of lactose dehydrogenase (LDH) indicating lytic cell death triggered by *S. pneumoniae* and accompanied by a reduction of IL-6 generation (Fig. 1C, D).

Given the findings that upregulation of neutrophil chemokines by human pericytes was by paracrine signalling from macrophages rather than by *S. pneumoniae* [25], we further examined the response of human brain pericytes to exposure to conditioned media obtained from THP-1 macrophages. Our protein array analysis showed a substantial increase in IL-6 release, but also a twofold higher release of CCL2, CCL3/4, G-CSF, and GM-CSF (Additional file 2: Figure S2). Furthermore, the stimulatory effect of conditioned medium from THP-1 macrophages exposed to *S. pneumoniae* was more than tenfold greater than that of *S. pneumoniae* alone, as shown by additional IL-6 ELISA analysis (231 ± 53 pg/ml and 2347 ± 174 pg/ml in human brain pericytes exposed to *S. pneumoniae* alone and conditioned medium from *S. pneumoniae*-stimulated THP-1 macrophages, respectively). When human brain pericytes were exposed to conditioned media from THP-1 cells lacking TLR2 or the inflammasome components ASC and Nlrp3, a strong reduction of IL-6 release could be observed. Similar results were obtained when human brain pericytes were challenged with conditioned media from WT THP-1 cells and treated with different inhibitors of the IL-1 signalling cascade, suggesting guidance of pericytes by neighbouring macrophages through IL-1β secretion (Fig. 1G, H). As pericytes can apparently adopt a macrophage-like phenotype under certain pathological conditions [41, 42], we further examined the expression of well-known macrophage markers, namely, CD11B, Cx3Cr1, Mrc1, CD36, CD163, and TREM2, in human pericytes, compared to the expression of PDGFRβ, without or after stimulation with conditioned medium. While PDGFRβ expression remained stable, we were unable to detect any expression of macrophage markers (data not shown).

### Pharmacological pericyte depletion resulted in a reduced survival time in the zebrafish model of PM

**Fig. 2:**
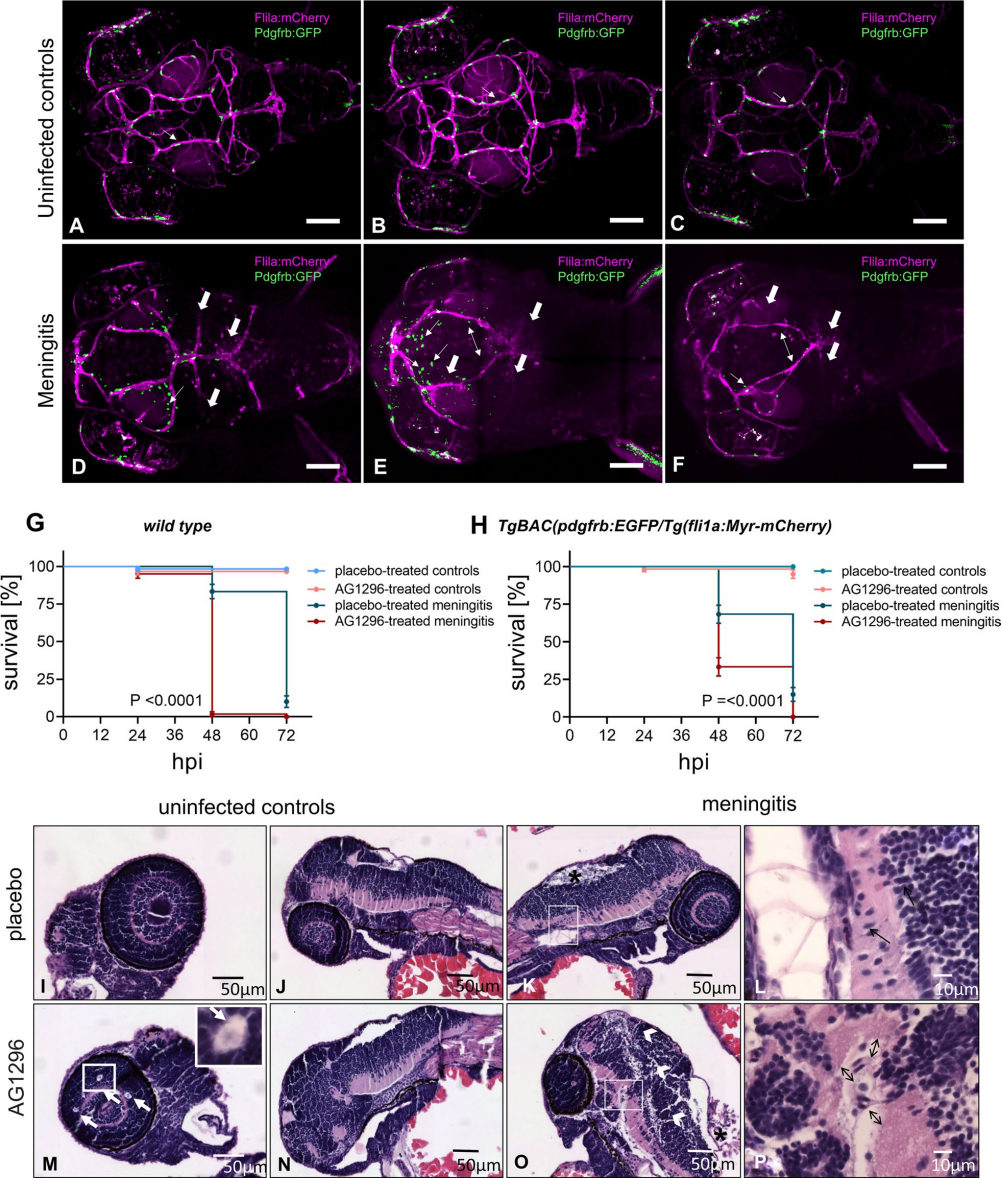
Confocal microscopy images from brains of *TgBAC(pdgfrb:EGFP/Tg(fli 1 a:Myr-mCherry)* transgenic zebrafish embryos that have had vehicle (uninfected controls, **A**–**C**) or *S. pneumoniae* (meningitis, **D**–**F**) injected into their hindbrain ventricles (three representative examples each). The embryos express eGFP in mural cells (namely, pericytes) and mCherry in endothelial cells. White bold arrows: compared to controls, infected embryos showed a marked reduction in mCherry-positive cells indicating meningitis-associated endothelial cell destruction (**D**–**F**). Thin double-headed arrows: a strong decrease in eGFP-positive cells was seen in some infected embryos, suggesting pericyte loss (**E**, **F**). Thin arrows: in others, there was predominantly a loss of contact between eGFP-positive and mCherry-positive cells (**D, E**). The scale bar indicates 100 μm length. (**A–F**). Effect of pharmacological pericyte depletion on the survival of wild-type (on the left) and *TgBAC(pdgfrb:EGFP/Tg(flila:Myr-mCherry*; on the right side*)* zebrafish embryos (**G**, **H**). Wild-type embryos were injected with 1900 cfu of live *S. pneumoniae* (= meningitis; or phenol red solution = controls; **G**); *TgBAC(pdgfrb:EGFP/Tg(flila:Myr-mCherry* embryos received 2200 cfu of live *S. pneumoniae* (= meningitis; or phenol red solution = controls, **H**). Embryos were treated either with DMSO as a placebo or the PDGFRβ inhibitor AG1296. Data are given as means ± SEM. Histopathological analysis of non-infected and *Streptococcus pneumonia*-infected wild-type zebrafish embryos treated either with DMSO as placebo (**I**–**L**) or the PDGFRβ inhibitor AG1296 (**M**–**P**) at 32 h post-injection (hpi). White arrows in **M**: embryos treated with AG1296 showed multiple cystic abnormalities in their eyes, compared to placebo-treated embryos (**I**). After *S. pneumoniae* infection, placebo- and AG1296-treated embroys showed signs of meningitis with presence of polymorphonuclear leukocytes in the ventricles (black asterisks) (**K**, **O**). White arrow heads in **O**: the brain parenchyma of AG1296-treated, infected embryos showed distinct edema, while the parenchyma of placebo-treated, infected embryos remained compact (**K**). Black arrows in **L, P**: the perivascular space was broadened in AG1296-treated embryos, compared to placebo-treated embryos

To examine the effects of *S. pneumoniae* on pericytes in vivo, we conducted experiments using transgenic zebrafish embryos expressing eGFP in pericytes and mCherry in endothelial cells [43]. By injecting *S. pneumoniae* or vehicle solution into the hindbrain ventricles of these embryos, we were able to observe changes in the pericyte population and their interaction with endothelial cells during PM. Confocal imaging of infected embryos 48 h after infection showed a marked reduction in mCherry-positive cells, indicating the destruction of endothelial cells and cerebral vessels due to meningitis (Fig. 2A–E). Infected embryos showed varying degrees of decrease in eGFP-positive pericytes or disruption in contact between pericytes and endothelial cells, suggesting disturbances in pericyte density and/or pericyte–endothelial cell interaction in the zebrafish PM model.

To gain insight into the functional role of pericytes in zebrafish PM, we pharmacologically depleted pericytes in WT and transgenic zebrafish embryos using the PDGFRβ inhibitor AG1296 [34, 44]. Confocal microscopic imaging confirmed the efficient depletion of pericytes AG1296-treated transgenic zebrafish embryos as compared to non-infected-placebo-treated embryos. At 48 h after the start of treatment, embryos that received AG1296 showed a marked reduction in GFP-positive cells compared to placebo-treated embryos (> 90%; which was paralleled by changes in the vasculature due to an impaired angiogenesis associated with pericyte loss) [45] (Additional file 2: Figure S2). When infected with *S. pneumoniae*, 50 out of 60 (83%) placebo-treated, but only 1 out of 60 (2%) embryos who received AG1296 survived the first 48 h. In contrast, the survival rates of control embryos injected with the control solution were 98% and 96% for placebo and AG1296 treatment (Fig. 2G, H). These findings indicate a detrimental effect of pericyte depletion on the survival of zebrafish embryos during PM.

Histopathological analyses of WT embryos harvested 48 h after ventricular injection revealed prominent brain edema formation in *S. pneumoniae-*infected embryos that were treated with the PDGFRβ inhibitor AG1296, while the brain parenchyma of placebo-treated, infected embryos looked similar to that found in non-infected controls, regardless of their treatment (Fig. 2I–P). In addition, in infected, AG1296-treated embryos, the perivascular spaces were regionally broadened compared to that of infected embryos, which were given the placebo (Fig. 2I–P).

### Genetic ablation of pericytes leads to worsening of disease in the mouse model of PM

**Fig. 3:**
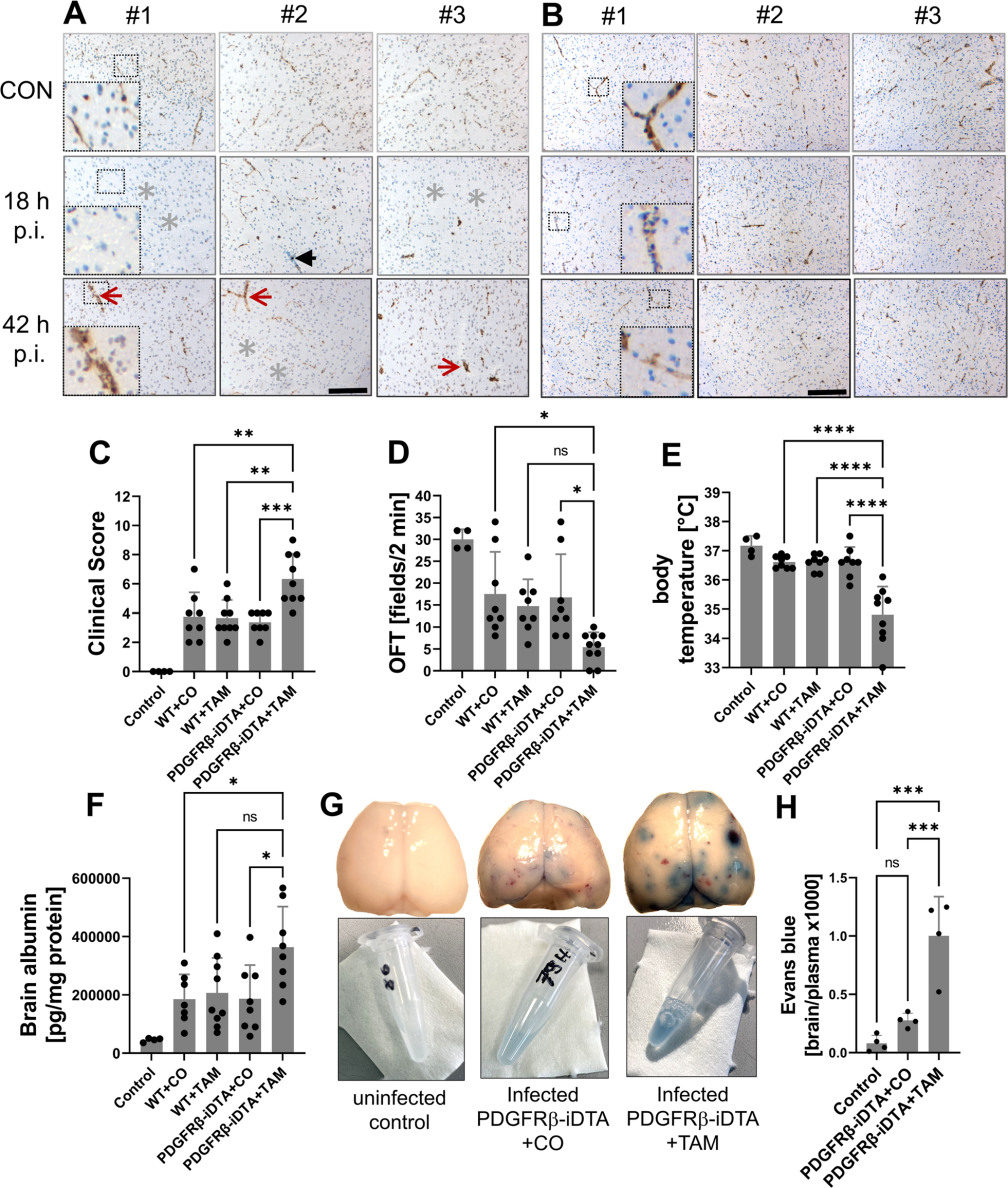
Regional changes in the immunoreactivity for PDGFRβ (**A**) and CD13 (**B**) in murine parietal cortices during PM. Brain sections were obtained from healthy controls (upper images) as well as infected mice at 18 and 42 h (h) after intracisternal application of *S. pneumoniae* (p.i. = post-infection; 3 mice per group, #1, #2, and #3; middle and lower images, respectively). The sections were stained either with anti-murine PDGFRβ or anti-murine CD13 antibodies and counterstained with hematoxylin–eosin. The scale bars on the lower middle images indicates 100 µm in length. PDGFRβ immunoreactivity appeared regionally disrupted (grey asterisks) and was missing at some vascular segments (black arrow), whereas in other vascular segments, PDGFRβ staining appears unchanged or even enhanced (red arrows). The determination of the disease phenotype 42 h after infection (**C**–**E**) showed a clear worsening of clinical symptoms in tamoxifen (TAM)-treated PDGFRB::creER2-iDTA mice (PDGFRβ-iDTA) which were largely missing pericytes, compared to corn oil (CO)-treated PDGFRB::creER2-iDTA mice (controls), as evidenced by significantly increased clinical score values (**E**), more restricted motor activities in the open-field test (OFT) (**D**), and lower body temperatures (**E**). The worsening of disease was associated with increased brain edema formation, as evidenced by increased brain albumin concentrations 42 h after infection (**F**) and enhanced Evans blue extravasation 24 h after infection (**G**, **H**) in TAM-treated PDGFRB::creER2-iDTA mice compared to controls. **G** Representative brain images taken immediately after perfusion and removal from one control mouse, one TAM- and one CO-treated PDGFRB::creER2-iDTA mouse (upper images) as well as the associated Evans blue extracts. Data are given as individual values as well as means ± SD. *P < 0.05, **P < 0.01, ***P < 0.001, ****P < 0.0001, using ANOVA with Tukey’s multiple comparisons test

To assess whether our observations made during embryonic development can be translated to the adult situation, we carried out further investigations in a mouse PM model [36, 40, 46]. First, we aimed to detect changes in the brain pericyte population associated with PM. For this we performed immunohistochemistry on brain sections obtained from mice before infection, as well as 18 and 42 h after infection using pericyte markers PDGFRβ and CD13 (Fig. 3A,B). Similar to the zebrafish model, we observed a heterogeneous staining pattern for both marker proteins in the mouse. Areas showing normal staining for PDGFRβ or CD13 were seen alongside areas of severely reduced staining (Fig. 3A,B).

Next, we used a genetic cell ablation model to gain insight into the importance of pericytes, more precisely of PDGFRβ-positive cells, in murine PM. Towards this aim we crossed the two previously used mouse strains PDGFRB::creER2 and R26-iDTA [39] to generate PDGFRB::creER2-iDTA mice and to induce depletion of PDGFRβ-expressing cells (namely, pericytes) by tamoxifen(TAM) application. [38]. PDGFRB::creER2-iDTA mice treated with corn oil—and also TAM- or corn oil-treated C57BL/6 mice—were applied as control groups. Immunohistochemical analysis of brain sections obtained from mice 42 h post-infection revealed a significant decrease in anti-PDGFRβ staining in TAM-treated PDGFRB::creER2-iDTA mice compared to those given corn oil, reflecting the efficacy of our cell depletion approach (Additional file 3: Figure S3). In contrast, staining with the fibroblast marker ER-TR-7 did not show any differences between TAM- and corn oil-treated transgenic mice (Additional file 3: Figure S3).

The determination of the disease phenotype 42 h post-infection showed a clear worsening of clinical symptoms in TAM-treated PDGFRB::creER2-iDTA mice compared to control groups, as evidenced by significantly increased clinical score values, more restricted motor activities, and lower body temperatures (Fig. 3C–E). The worsening of disease was neither associated with differences in blood and brain bacterial titres nor with the extent of cerebral haemorrhages, a surrogate marker of brain pathology in adult meningitis mice, but also in patients who died from PM [46, 47] (Additional file 4: Figure S4). However, TAM-treated PDGFRB::creER2-iDTA mice exhibited significantly increased brain albumin concentrations, which is a well-accepted indicator of BBB breaching [40], as compared to control groups (Fig. 3F). To further substantiate this finding, we performed additional experiments in which mice were given Evans blue dye 2 h before the end of the experiment (which in this case was 24 h post-infection). Evans blue is widely used to monitor vascular protein leakage [48]. Brain Evans blue concentrations were significantly higher in TAM-treated than in corn oil-treated PDGFRB::creER2-iDTA mice. The visual inspection of the brains (before Evans blue extraction) revealed patches of the dye, which is surprising, since the depletion approach affected the entire brain. The distribution of Evans blue extravasation did not overlap with cerebral bleeding foci (Fig. 3G,H).

**Fig. 4:**
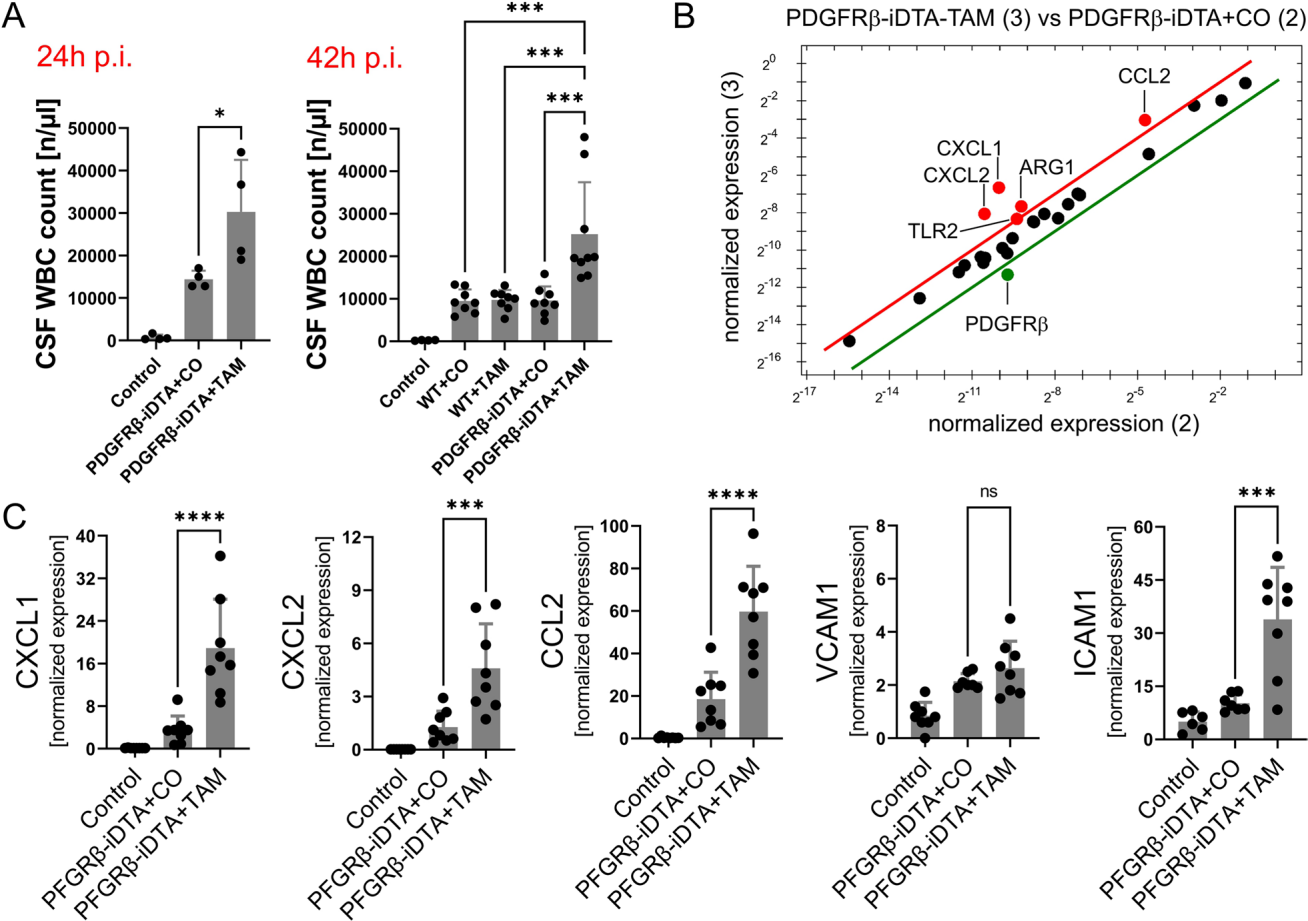
Cerebrospinal (CSF) white blood cell (WBC) counts at 24 and 42 h post-infection in TAM-treated PDGFRB::creER2-iDTA mice (PDGFRβ-iDTA) that are largely missing pericytes compared to control groups (**A**). Prime™ PCR array analysis of cDNA obtained from brains of infected transgenic mice revealed higher expression levels of CXCL1, CXCL2, and CCL2 (and also of TLR2 and ARG1), but lower PDGFRβ expression following pericyte depletion (**B**). The array results were confirmed by supplemental RT-PCR analyses (**C**), which showed increased brain expression of CXCL1, CXCL2, and CCL2 in TAM-treated PDGFRB::creER2-iDTA mice. Data are given as individual values as well as means ± SD. *P < 0.05, **P < 0.01, ***P < 0.001, ****P < 0.0001, using ANOVA with Tukey’s multiple comparisons test

In addition to the more pronounced BBB disruption, infected, TAM-treated PDGFRB::creER2-iDTA mice had higher CSF white blood cell (WBC) counts (both 24- and 42-h postinfection) compared to control mice (Fig. 4A). This finding was unexpected and did not align with previous in vitro findings [25]. To explore possible explanations, we conducted a Prime™ PCR array analysis of cDNA obtained from brains of infected TAM-treated vs corn oil-treated transgenic mice. TAM-treated PDGFRB::creER2-iDTA mice showed elevated expression levels of chemokines CXCL1, CXCL2, and CCL2 (as well as TLR2 and ARG1), along with lower PDGFRβ expression compared to corn oil-treated transgenic mice (Fig. 4C). Based on previous investigations linking pericyte depletion to increased leukocyte migration and elevated levels of ICAM-1 and VCAM-1 [49–51], we further investigated their mRNA expression values. We found that brain ICAM-1 levels were increased in TAM-treated PDGFRB::creER2-iDTA mice compared to CO-treated PDGFRB::creER2-iDTAmice, whereas VCAM-1 levels were unchanged (Fig. 4C).

## Discussion

We show that PM induces notable alterations within the population of brain pericytes. Experimentally induced depletion of pericytes exacerbated the progression of the disease, leading to increased migration of leukocytes from the bloodstream into the CSF, along with severe disruption of the BBB. Thus, preserving the integrity of the pericyte population poses a new therapeutic strategy in PM.

Recruitment of leukocytes, in particular of neutrophils, into the CSF and brain is a key hallmark of PM [46, 47]. Several in vitro studies have shown that, through direct interactions mediated by cytokines, chemokines, and/or ICAM-1, cultured pericytes can promote neutrophilic migration by providing ‘tracks’ for the neutrophils to migrate along, by directing their movement along chemical gradients, and by increasing their activation state and survival [15, 18, 25, 52, 53]. Supporting observations were made in vivo in peripheral organs. For example, during cytokine (e.g., IL-1β)-induced inflammation in the murine cremaster muscle, neutrophils were found to be guided via pericyte ICAM-1 to pericyte gaps to overcome the vessel wall and enter the tissue [54]. In murine inflamed ear skin, pericytes on capillaries and arterioles were shown to aid chemotactic immigration into the interstitium by interacting with neutrophils via ICAM-1, and further support the migratory response of neutrophils by providing a set of chemokines [52]. Surprisingly, while pericytes appear to enable or at least promote leukocyte recruitment into peripheral organs, their absence seems to render the CNS vasculature permissive to neutrophil emigration. Torök and colleagues [50] showed that adult pericyte-deficient mice (Pdgfβ^ret/ret^ mice) exhibited increased leukocyte infiltration (mainly monocytes, dendritic cells, and T cells) into the CNS both during homeostasis and upon induction of experimentally autoimmune encephalitis. This increased immigration of leukocytes appears to be largely due to a pro-inflammatory phenotype of endothelial cells, characterized by increased expression of ICAM-1, VCAM-1, and CCL2, that can be observed in the absence of pericytes [49–51]. In agreement with and as a complement to these studies, we show here that the absence of pericytes leads to significantly increased leukocyte migration into the CNS during an acute bacterial infection, which is associated with an increased brain expression of the neutrophil chemokines CXCL1 and CXCL2 as well as ICAM-1 (and the monocyte chemoattractant CCL2). Thus, pericytes appear to have a different function in the CNS than in peripheral organs and limit leukocyte migration, presumably by counteracting the production of leukocyte adhesion molecules and chemokines.

The massive and long-term accumulation of neutrophils is considered to be a decisive pathogenetic factor in meningitis-associated vascular damage [1, 2]. Therefore, it is not surprising that the increased leukocyte infiltration was accompanied by more pronounced BBB dysfunction and brain edema formation in our animal models of PM when pericytes were absent. This finding is all the less surprising, since there is now sufficient evidence that brain pericytes alone make a decisive contribution to the integrity of the BBB (e.g., among others by promoting the expression of tight junction proteins) [20, 49, 55, 56]. Conversely, a coincidence of pericyte loss and BBB disruption has been demonstrated in various neurological disorders, such as stroke, traumatic brain injury, amyotrophic lateral sclerosis or Alzheimer’s disease [57–60, 60–62]. Furthermore, in an *Escherichia coli*–lipopolysaccharide septic encephalopathy model, pericyte detachment from the basal lamina in the hippocampus was observed [63]. This detachment was closely associated with increased cerebrovascular permeability due to the disarray in the pericyte and basal lamina [64]. Consistent with these findings, our investigations revealed substantial alterations in the pericyte population during the progression of meningitis, including regional cell loss. In addition, induced depletion of pericytes, both pharmacologically and genetically, in two animal models of meningitis resulted in increased breach of the BBB and brain edema. Intriguingly, when examining the brains, only a spot-like leakage of the peripherally introduced dye Evans blue was found both in control and pericyte-depleted animals, even though the genetic depletion led to a homogeneous reduction in pericytes throughout the cerebral cortex. This observation suggests that pericyte loss may exacerbate meningitis-associated BBB disruption, but is not solely responsible for this damage.

In meningitis, vascular damage may result from a direct attack by the pathogens (namely, bacterial toxins, such as pneumolysin) or host-derived factors, including oxidants and proteolytic enzymes [1, 65–67]. Regardless of this, it seems worth considering to test the effect of therapeutic measures counteracting pericyte damage in the context of meningitis, since our experiments showed both a disturbance of the pericyte population in meningitis and a drastic clinical deterioration after induced pericyte depletion. An approach that would deserve further investigation is a local or systemic administration of the growth factor PDGFRB, which is critical for the maintenance of pericyte vessel coverage and normal BBB function [68] and has already been tested in experimental models of status epilepticus or Parkinson’s disease with positive results [69].

Our study has several limitations. First, although PDGFRβ is a widely used pericyte marker, it is not highly specific. PDGFRβ is also expressed on other cell types in the CNS, for example, vascular smooth muscle cells and perivascular fibroblasts [70]. As far as fibroblasts are concerned, we were able to show by immunohistochemistry that the genetic depletion approach used had no effect on the staining pattern for ER-TR-7, an established fibroblast marker (supplemental material) [71, 72]. In the zebrafish model, we were able to identify pericytes based on the combination of PDGFRβ expression and their morphology and location on the vessel using confocal microscopy. Second, although we were able to show a clear connection between the increased leukocyte migration and the increased expression of selected chemokines in the brain of pericyte-depleted mice, it remains unclear which cell types express these chemokines. Possible candidates could be resident macrophages. Third, we provided evidence that the loss of pericytes resulted in a significant increase in meningitis-associated BBB permeability. Nevertheless, the validation of structural alterations in the BBB, such as modifications in endothelial tight junctions or endothelial–astrocyte interactions, and the elucidation of the underlying mechanisms necessitate further investigations in subsequent studies. For example, the question as to whether the increased leukocyte immigration is responsible for this phenomenon still needs to be addressed. Fourth, we used the model most commonly employed in meningitis research, in which the pathogens are directly injected into the cisterna magna. This model reflects direct infection from a neighboring infectious focus but does not mimic the hematogenous origin of meningitis. To analyze the role of pericytes in bacterial traversal of the BBB in hematogenous meningitis, additional experiments using an intranasal or intravenous infection model are required. Fifth, in the mouse experiments only animals surviving the full 42 h were assessed for clinical scores, CSF white blood cell counts, bacterial titer, and brain pathology. As deceased mice can be assumed to be more severely affected on these parameters, clinical and pathological differences between pericyte-ablated and control mice may have been underestimated or even missed as numbers of surviving animals were too small to show statistically significant differences. Finally, our cell-culture experiments involving a PLY-deficient D39 strain provided preliminary indications of a potential involvement of this bacterial toxin in cytokine expression by pericytes. This finding warrants validation through subsequent investigations utilizing purified PLY and/or PLY variants (such as those lacking the pore-forming domain).

In conclusion, our data suggests a significant role of pericytes in the pathogenesis of PM, especially in the regulation of leukocyte recruitment and maintenance of BBB integrity. Future investigations should explore the efficacy of pericyte-targeted therapeutics as adjuncts to antibiotics for PM.

### Supplementary Information

**Additional file 1: Figure S1.** Interleukin (IL)-6 and lactate dehydrogenase (LDH) release from human (green and orange bars) and murine (blue and red bars) primary brain pericytes after exposure to increasing concentrations (MOI = 2.5, 10, 40, 160) of the pneumococcal serotypes 7F (7F), 6B (6B), 2 (D39), as well as a pneumolysin-deficient isogenic D39 mutant (D39ΔPLY). Data from serotype 2 (grey bars) are also shown in Fig. 1. Data are given as individual values as well as means ± SD. * *P* < 0.05, ** *P* < 0.01, *** *P* < 0.001, **** *P* < 0.0001, using ANOVA with Tukey’s multiple comparisons test.

**Additional file 2: Figure S2.** Optical densities of the protein spots (cytokines) that were displayed on the protein array membranes. Density determination after inverting the digitized chemiluminescence images using the Image J software. HBVP = human brain vascular pericytes, CM = conditioned medium, THY = Todd–Hewitt broth supplemented with yeast extract. Sp = *S. pneumoniae*.

**Additional file 3: Figure S3.** Confocal microscopy images of two representative, uninfected *TgBAC(pdgfrb:EGFP/Tg(flila:Myr-mCherry* embryos (96 h post-fertilization; hpf) treated with either DMSO as placebo (A) or the PDGFRβ inhibitor AG1296 (B) for 48 h. In placebo-treated embryos, numerous GFP-positive cells (indicative for pericytes) can be seen which co-localize with blood vessels (thin white arrows). AG1296 treatment resulted a marked reduction of GFP-positive cells (thick white block arrows); only a few cells that are in contact with blood vessels remain (white arrow heads). Scale bars, 100 µm.

**Additional file 4: Figure S4.** Immunoreactivity for PDGFRβ (A) and ER-TR7 (B) in murine brains during PM. A. Brain sections were obtained from infected TAM- or CO-treated PDGFRB::creER2-iDTA mice (PDGFRβ-iDTA) 42 h after intracisternal application of *S. pneumonia*e (two representative examples per group: #1 and #2). Three randomly selected section per animal are shown. The sections were stained with an anti-murine PDGFRβ antibody and counterstained with hematoxylin–eosin. A marked reduction in PDGFRβ staining was observed in TAM-treated transgenic mice compared to the CO-treated animals, suggesting successful depletion of PDGFRβ-positive cells, presumably pericytes. B. Brain sections were obtained from uninfected wild-type control mice, PDGFRB::creER2-iDTA mice treated with CO, and PDGFRB::creER2-iDTA mice treated with TAM 42 h after intracisternal application of *S. pneumonia*e (one representative example per group). The sections were stained with an anti-murine ER-TR7 antibody and weakly counterstained with hematoxylin–eosin. There was no visible reduction or difference in ER-TR7 immunoreactivity between the experimental groups, which suggests preservation of the CNS fibroblast population in this genetic cell ablation model.

**Additional file 5: Figure S5.** Cerebellar bacterial concentrations (titers; **A**) and the number of cerebral bleeding foci (**B**) 42 h after intracisternal injection of either phosphate-buffered saline (control) or 10^5^ colony forming units (cfu) *S. pneumoniae* (all other experimental groups). (**C**) Exemplary photos of mouse brains (one per experimental group) obtained 42 h after infection and cardiac perfusion and thereafter placed in freezing medium.

**Additional file 6: Supplemental table**. List of elements on the customized PrimePCR Array.

## Data Availability

The data sets used and/or analysed during the current study are available from the corresponding author on reasonable request.

## Abbreviations

ASC: Apoptosis-associated speck-like protein containing a caspase recruitment domain
BBB: Blood–brain barrier
CCL2: C–C motif chemokine ligand 2
CNS: Central nervous system
CSF: Cerebrospinal fluid
CXCL1: C–X–C motif chemokine ligand 1
EB: Evans Blue
ICAM-1: Intercellular adhesion molecule-1
LDH: Lactate dehydrogenase
NF-κB: Nuclear factor ‘kappa-light-chain-enhancer’ of activated B cells
Nlrp3: NOD-, LRR- and pyrin domain-containing protein 3
PAMPs: Pathogen-associated molecular patterns
PM: Pneumococcal meningitis
PRRs: Pattern recognition receptors
TAM: Tamoxifen
*Tg*: *TgBAC(pdgfrb:EGFP/Tg(fli1a:Myr-mCherry)*
THP-1: Tohoku Hospital Pediatrics-1
TLRs: Toll-like receptors
WBC: White blood cell
Wt: Wild type
T2.5: Antibody directed against TLR2
Hpf: Hour postfertilization
Dpf: Days postfertilization
DMSO: Dimethylsulfoxid
OFT: Open-field test
