# Supplementary figures and images for: Pericytes are protective in experimental pneumococcal meningitis through regulating leukocyte infiltration and blood–brain barrier function
Source: J Neuroinflammation. 2023 Nov 17;20:267. doi: 10.1186/s12974-023-02938-z (PMC10655320; doi:10.1186/s12974-023-02938-z)

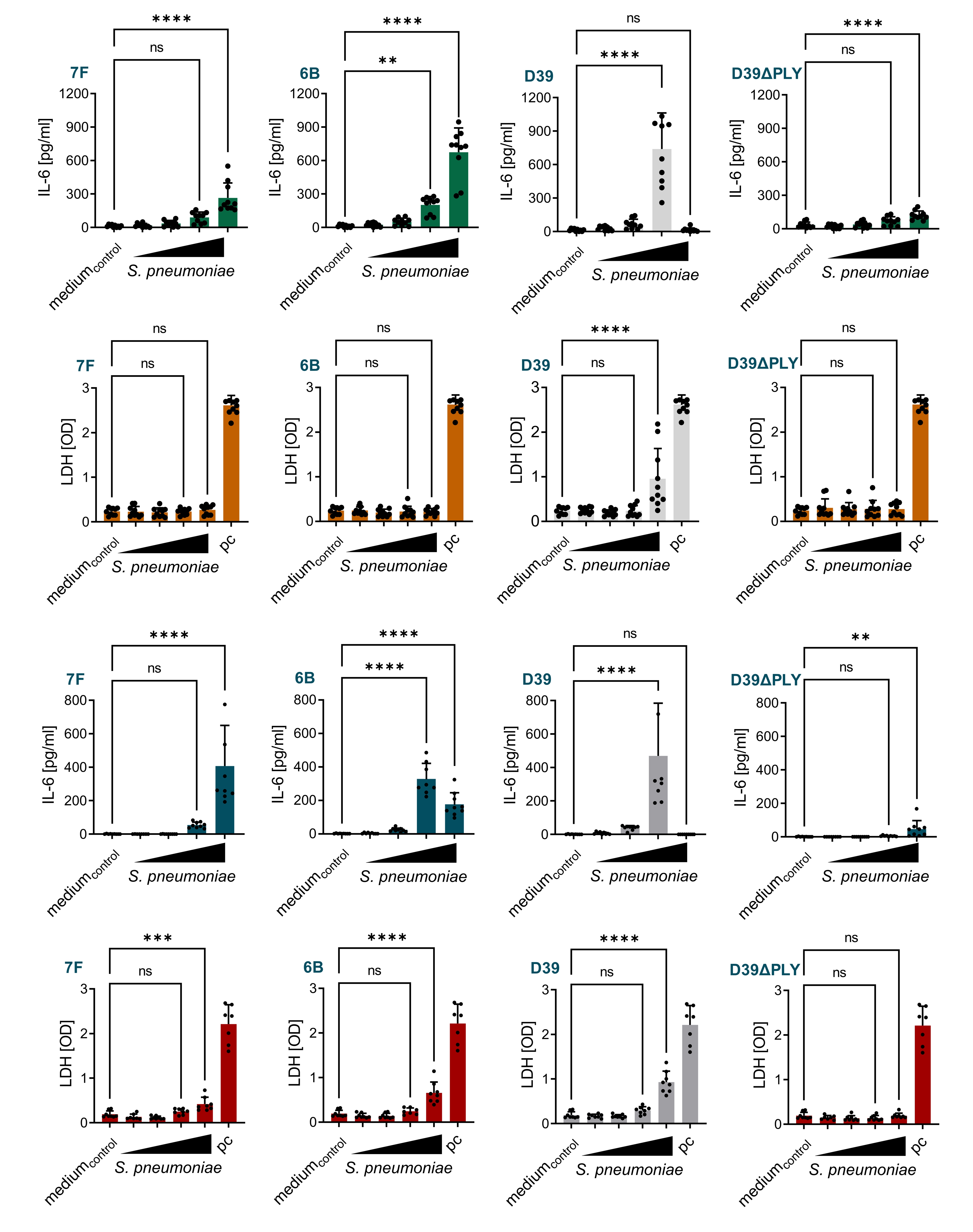

Supplement: Supplementary file 1 — Additional file 1: Figure S1. Interleukin (IL)-6 and lactate dehydrogenase (LDH) release from human (green and orange bars) and murine (blue and red bars) primary brain pericytes after exposure to increasing concentrations (MOI = 2.5, 10, 40, 160) of the pneumococcal serotypes 7F (7F), 6B (6B), 2 (D39), as well as a pneumolysin-deficient isogenic D39 mutant (D39ΔPLY). Data from serotype 2 (grey bars) are also shown in Fig. 1. Data are given as individual values as well as means ± SD. * P < 0.05, ** P < 0.01, *** P < 0.001, **** P < 0.0001, using ANOVA with Tukey’s multiple comparisons test. [file 12974_2023_2938_MOESM1_ESM.jpg]

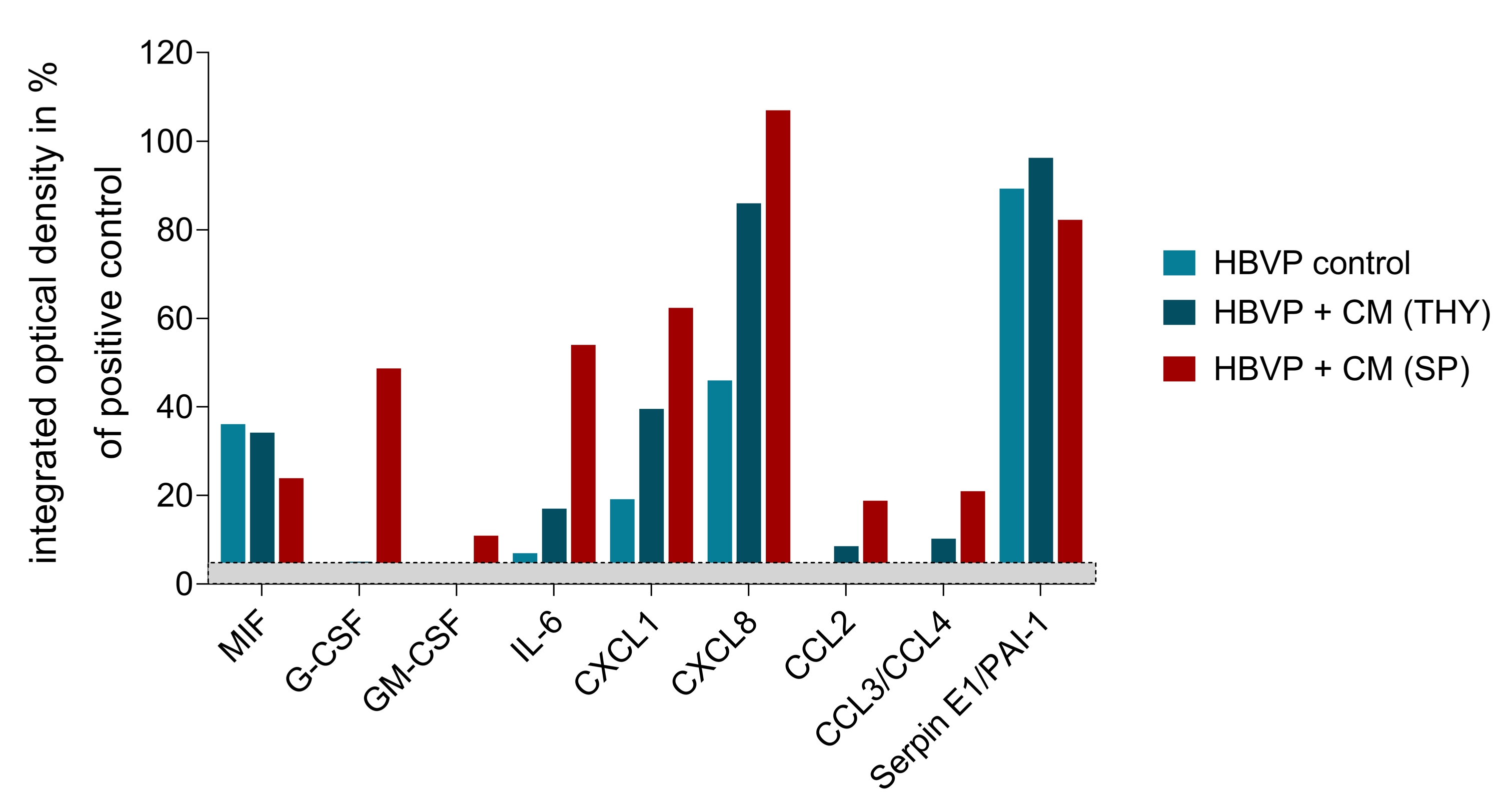

Supplement: Supplementary file 2 — Additional file 2: Figure S2. Optical densities of the protein spots (cytokines) that were displayed on the protein array membranes. Density determination after inverting the digitized chemiluminescence images using the Image J software. HBVP = human brain vascular pericytes, CM = conditioned medium, THY = Todd–Hewitt broth supplemented with yeast extract. Sp = S. pneumoniae. [file 12974_2023_2938_MOESM2_ESM.jpg]

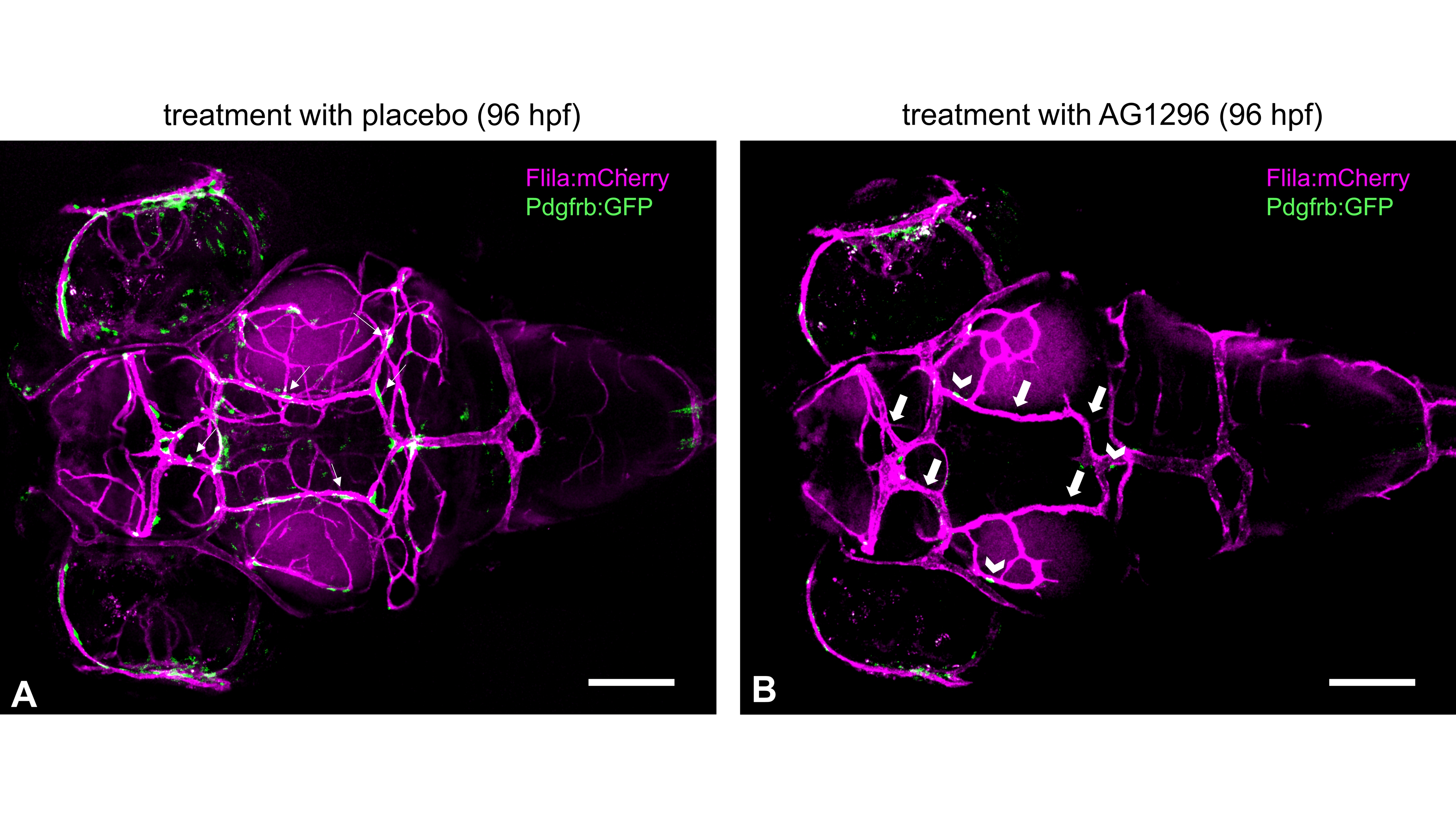

Supplement: Supplementary file 3 — Additional file 3: Figure S3. Confocal microscopy images of two representative, uninfected TgBAC(pdgfrb:EGFP/Tg(flila:Myr-mCherry embryos (96 h post-fertilization; hpf) treated with either DMSO as placebo (A) or the PDGFRβ inhibitor AG1296 (B) for 48 h. In placebo-treated embryos, numerous GFP-positive cells (indicative for pericytes) can be seen which co-localize with blood vessels (thin white arrows). AG1296 treatment resulted a marked reduction of GFP-positive cells (thick white block arrows); only a few cells that are in contact with blood vessels remain (white arrow heads). Scale bars, 100 µm. [file 12974_2023_2938_MOESM3_ESM.jpg]

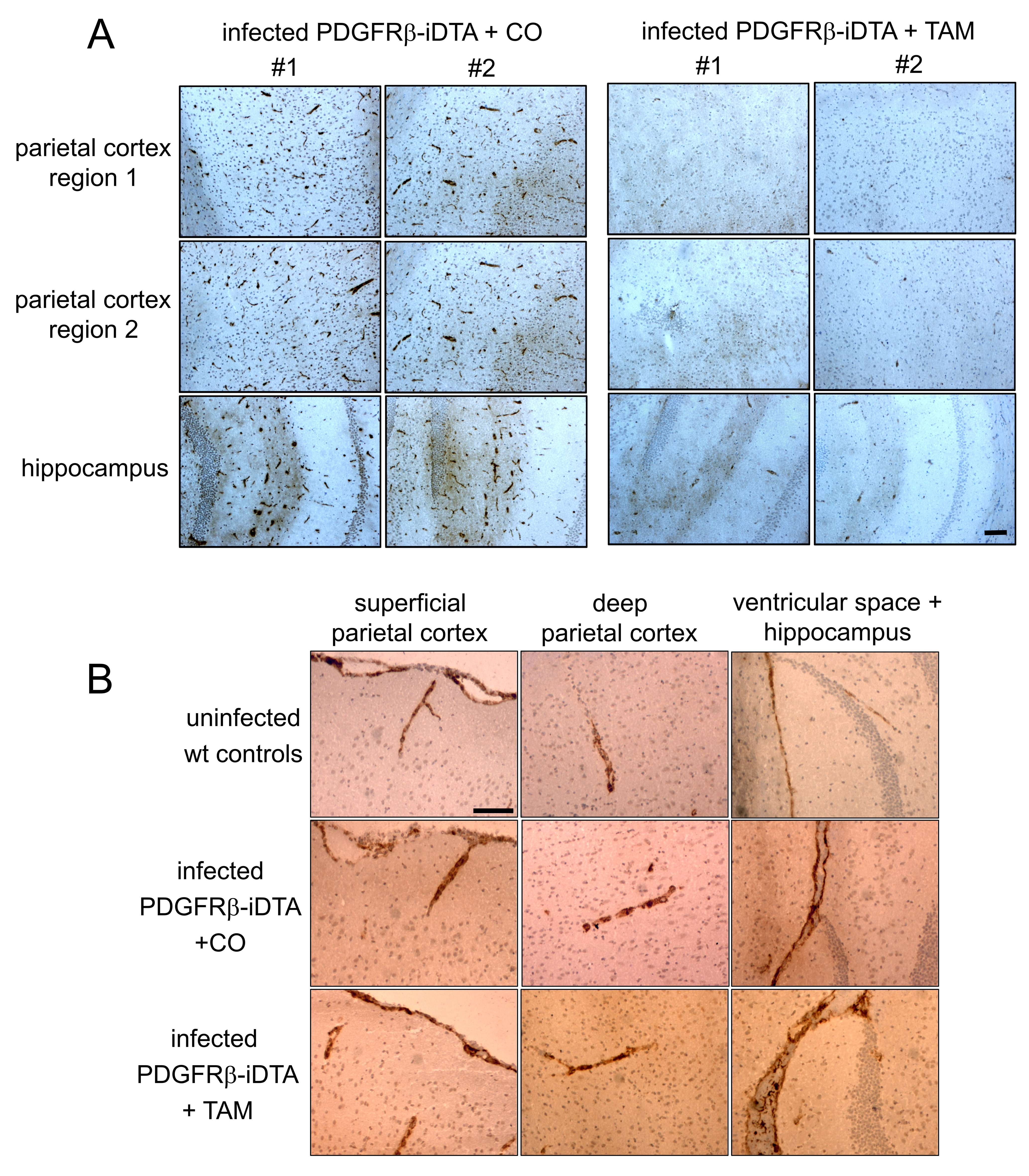

Supplement: Supplementary file 4 — Additional file 4: Figure S4. Immunoreactivity for PDGFRβ (A) and ER-TR7 (B) in murine brains during PM. A. Brain sections were obtained from infected TAM- or CO-treated PDGFRB::creER2-iDTA mice (PDGFRβ-iDTA) 42 h after intracisternal application of S. pneumoniae (two representative examples per group: #1 and #2). Three randomly selected section per animal are shown. The sections were stained with an anti-murine PDGFRβ antibody and counterstained with hematoxylin–eosin. A marked reduction in PDGFRβ staining was observed in TAM-treated transgenic mice compared to the CO-treated animals, suggesting successful depletion of PDGFRβ-positive cells, presumably pericytes. B. Brain sections were obtained from uninfected wild-type control mice, PDGFRB::creER2-iDTA mice treated with CO, and PDGFRB::creER2-iDTA mice treated with TAM 42 h after intracisternal application of S. pneumoniae (one representative example per group). The sections were stained with an anti-murine ER-TR7 antibody and weakly counterstained with hematoxylin–eosin. There was no visible reduction or difference in ER-TR7 immunoreactivity between the experimental groups, which suggests preservation of the CNS fibroblast population in this genetic cell ablation model. [file 12974_2023_2938_MOESM4_ESM.jpg]

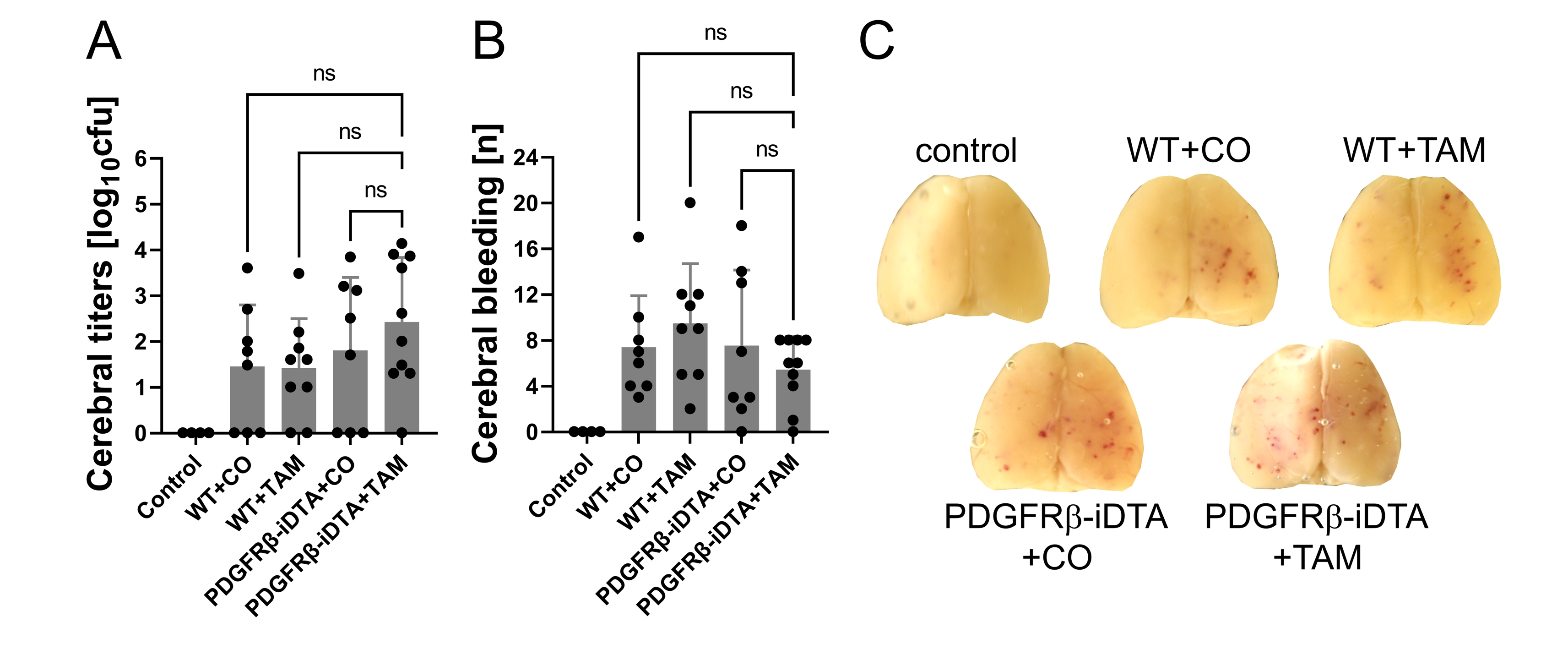

Supplement: Supplementary file 5 — Additional file 5: Figure S5. Cerebellar bacterial concentrations (titers; A) and the number of cerebral bleeding foci (B) 42 h after intracisternal injection of either phosphate-buffered saline (control) or 105 colony forming units (cfu) S. pneumoniae (all other experimental groups). (C) Exemplary photos of mouse brains (one per experimental group) obtained 42 h after infection and cardiac perfusion and thereafter placed in freezing medium. [file 12974_2023_2938_MOESM5_ESM.jpg]
