# Supplementary material for: Pericytes are protective in experimental pneumococcal meningitis through regulating leukocyte infiltration and blood–brain barrier function
Source: J Neuroinflammation. 2023 Nov 17;20:267. doi: 10.1186/s12974-023-02938-z (PMC10655320; doi:10.1186/s12974-023-02938-z)
Supplement: Supplementary file 6 — Additional file 6: Supplemental table. List of elements on the customized PrimePCR Array. [file 12974_2023_2938_MOESM6_ESM.pdf]

Supplemental table: list of elements on the customized PrimePCR Array

Gene code	Protein
Aim2	AIM2, absent in melanom 2
Anpep	aminopepidase N, CD13, etc.
Arg1	arginase 1
Casp1	CASP1, caspase 1
Ccl2	CCL2 , C-C motif chemokine 2
Cd163	CD163, scavenger receptor cysteine-rich type 1 protein M130
Cd36	CD36, platelet glycoprotein 4, etc.
Col1A1	Col1A1, collagen alpha 1 chain
Cxcl1	CXCL1, growth-regulated alpha protein, C-X-C motif chemokine 1
Cxcl2	CXCL2, C-X-C motif chemokine 2, growth-regulated protein beta
Gfap	GFAP, glial fibrillary acidic protein
Il10	IL-10, interleukin-10
Il18	IL-18, interleukin-18
Il1b	IL-1β, interleukin-1beta
Il6	IL-6, interleukin-6
Itgam	ITGAM, integrin subunit alpha M, CD11B
Mrc1	MRC1, macrophage mannose receptor 1, CD206
Nanos2	NOS2, nitric oxide synthase 2
Nefl	NEFL, neurofilament light polypeptide
Nlrp3	NLRP3, NACHT, LRR and PYD domains-containing protein 3
Pdgfra	PDGFRα, platelet-derived growth factor receptor alpha
Pdgfrb	PDGFRβ, platelet-derived growth factor receptor alpha
Tlr13	TLR13, Toll-like receptor 13
Tlr2	TLR2, Toll-like receptor 2
Tlr4	TLR4, Toll-like receptor 4
*Actb**	ActB, actin, cytoplasmic 1, beta-actin
*Gapdh**	GAPDH, glyceraldehyde-3-phosphate dehydrogenase

* housekeeping genes
